# Pancreatic mucinous cystic neoplasm with sarcomatous stroma metastasizing to liver: a case report and review of literature

**DOI:** 10.1186/1477-7819-11-100

**Published:** 2013-05-20

**Authors:** Michael Wayne, Deniz Gur, Gil Ascunce, Ben Abodessa, Violette Ghali

**Affiliations:** 1 Biliary and Pancreatic Surgery of New York, Beth Israel Medical Center, New York, USA

## Abstract

We report a case of mucinous cystic neoplasm of pancreas with sarcomatous stroma metastasizing to the liver. The tumor occurred in a male patient aged 46 years. Symptoms included persistent epigastric and right upper quadrant pain. Radiographically, the pancreas contained four large cystic masses located in the neck, body, and tail. Histologically, the cysts were lined with benign, mucinous epithelium with underlying bland, storiform, ovarian-like stroma. An undifferentiated focally hyalinized, sarcomatous stroma composed of bland spindle cells showing short fascicular growth pattern and focal nuclear palisading was associated with the epithelial component in one of the cysts. These cells showed strong immunoreactivity with vimentin and inhibin (weak), they were negative for CD34, estrogen receptor, progesterone receptor, androgen, calretinin, S-100, CD117, melan A, chromogranin, and synaptophysin. A morphologically and immunohistochemically identical metastatic sarcomatous focus was identified in the liver without any glandular component. This case is unique in its clinically malignant behaviour and metastatic nature despite its morphologically benign epithelial and stromal components.

## Background

Mucinous cystic neoplasms (MCNs) represent one of the major cystic neoplasms of the pancreas. They have distinct clinicopathological features. They are seen in women in the fifth or sixth decades of life; only rarely are examples documented in men. The tumor is usually located in the tail of the pancreas. Macroscopically, the cysts are multilocular and they do not communicate with the pancreatic ductal system. Histologically, the lining epithelium consists of tall, columnar cells with abundant apical mucin, although cuboidal cells that lack obvious mucin may also be present. The epithelium may be bland in appearance or might range from mild to severe atypia resembling ovarian counterparts and include mucinous cystadenomas, borderline mucinous cystic tumors, and mucinous cystadenocarcinomas. Another distinct histologic feature is the presence of subepithelial hypercellular, ovarian-like spindle cell stroma, and its presence has become a requirement for the diagnosis of this entity [1,2]. Here we report a case of mucinous cystic neoplasm of the pancreas with sarcomatous stroma metastasized to liver.

### Case presentation

**Figure 1:**
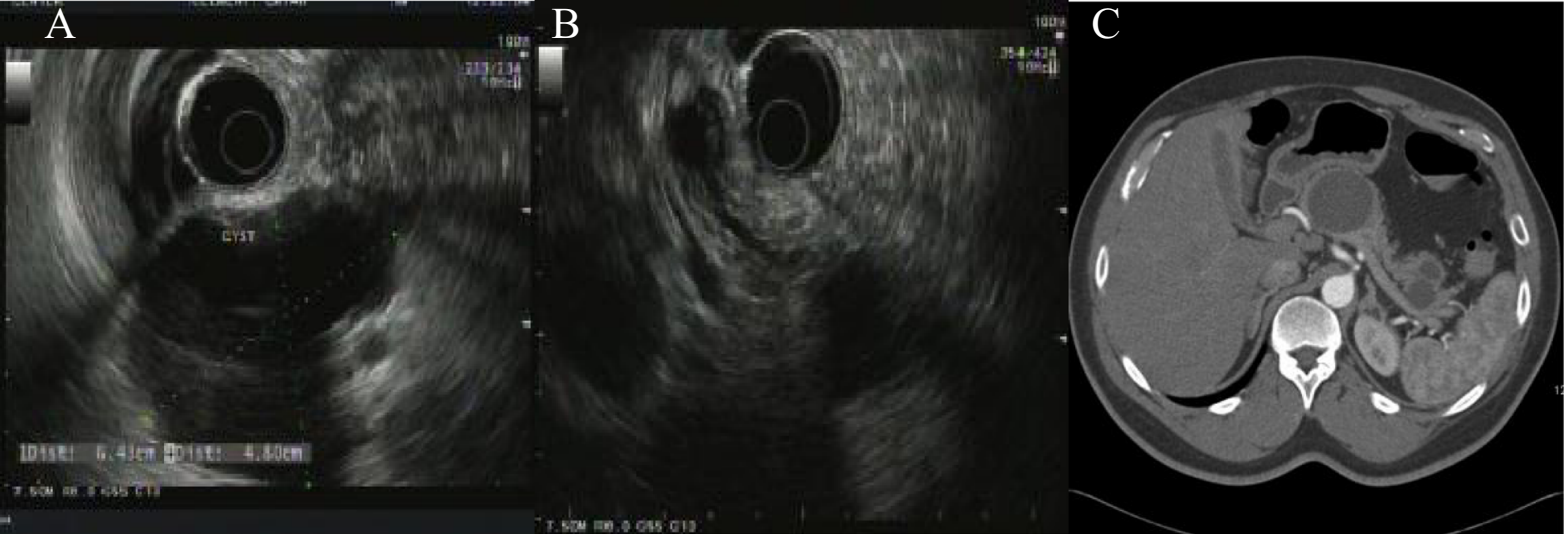
**Endoscopic ultrasonography and computed tomography. A-B**) Endoscopic ulrasonography showing cysts in neck, body and tail of Pancreas. **C**) Computed tomography of pancreas showing cystic mass with hemorrhage.

A 46-year-old man presented with persistent epigastric and right upper quadrant pain. Endoscopic ultrasound studies revealed multiple cysts in the pancreas (Figures 1A,B). Computed tomography (Figure 1C) of the abdomen showed a large mass in the neck and body of the pancreas measuring 6.8 × 5.7 cm in cross-section, consistent with a cystic mass with internal hemorrhage. In addition, a 1.2 cm cystic mass in the pancreatic body and three adjacent cystic masses in the pancreatic tail measuring 2.3, 1.7, and 1.4 cm were identified. No pancreatic ductal dilatation was seen.

### Pathologic findings

**Figure 2:**
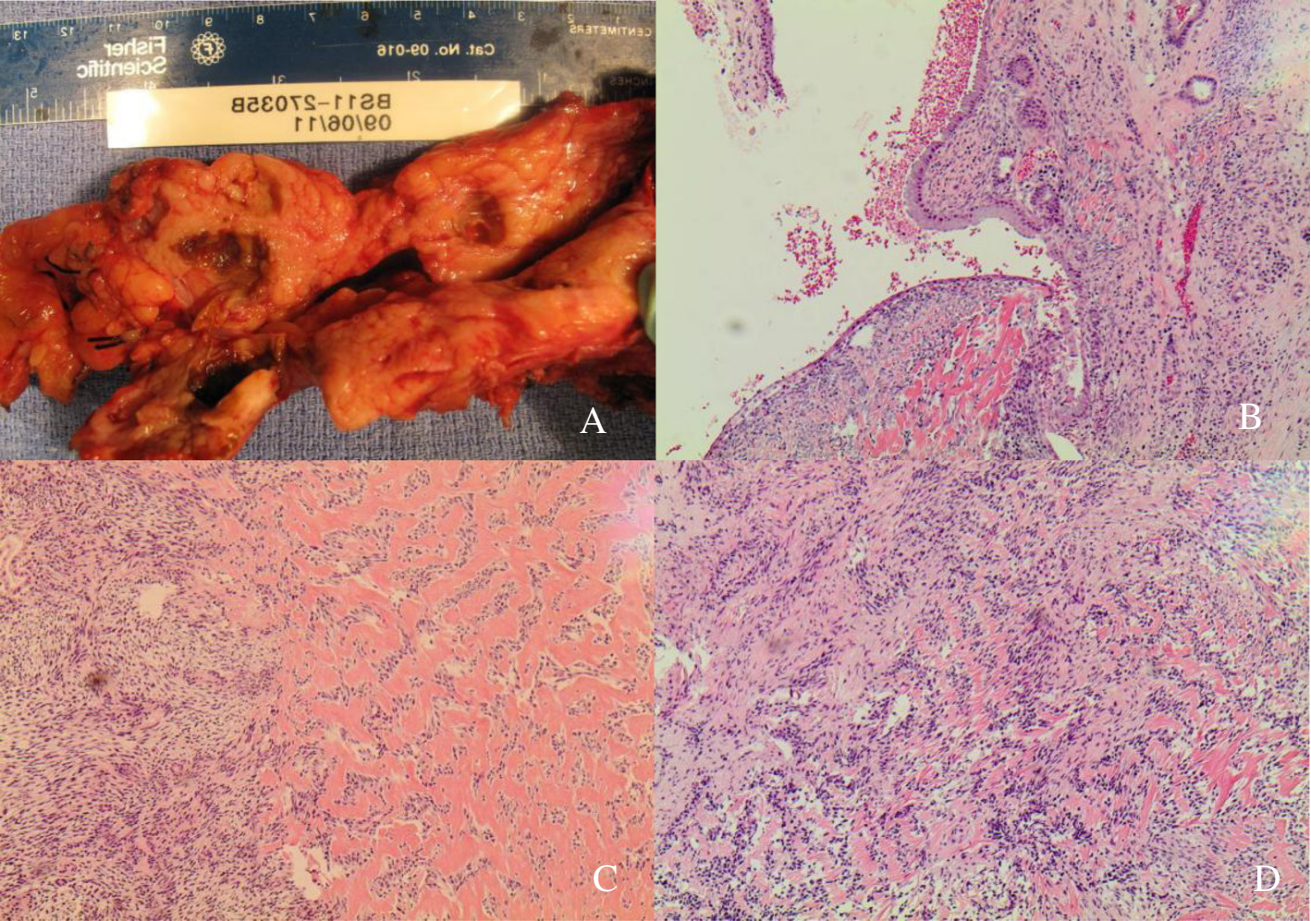
**Pictures of pancreatic cysts, cyst lined by benign mucinous epithelium, sarcomatous stroma in pancreas, and sarcomatoid metastatic foci in liver.** (**A**) Gross picture of pancreatic cysts occupying neck, body and tail. (**B**) Cyst lined by benign mucinous epithelium with underlying ovarian-like, storiform stroma (H & E, 10×) (**C**) Sarcomatous stroma in pancreas. Spindle stromal cells with fascicular growth pattern and nuclear palisading. Focal areas of dense, acellular, hyalinized tissue are evident (H & E, 20×) (**D**) Sarcomatoid metastatic foci in liver. Morphology is identical to lesion in pancreas (H & E, 20×).

A partial pancreatectomy removed four cysts ranging in size from 1.5 × 1.5 × 0.8 cm to 7.0 × 5.0 × 3.5 cm, located in the neck, body, and tail of the pancreas. All the cysts had a green-tan to red, smooth, glistening lining. The parenchyma separating the cysts was tan-yellow and focally indurated (Figure 2A).

Histologically, the cysts were lined with columnar cells with mucinous laden cytoplasm and basally located, bland nucleus. Beneath the epithelial lining, a hypercellular ovarian-like stroma composed of spindle-shaped cells in a storiform growth pattern was observed (Figure 2B). In some areas, the spindle cells had a fascicular growth pattern and focally nuclear palisading was evident. In these areas, the cells had mild atypia; however, no increase in mitotic figures was detected. The focal areas of the stroma were composed of dense, acellular, hyalinized tissue (Figure 2C). A liver segment resected at the same time revealed a metastatic stromal lesion composed entirely of spindle-shaped cells identical to those seen in the pancreas but without the glandular component (Figure 2D). The uninvolved pancreas showed fibrosis and atrophy. All of the 11 lymph nodes were free of metastasis.

### Immunohistochemistry

**Figure 3:**
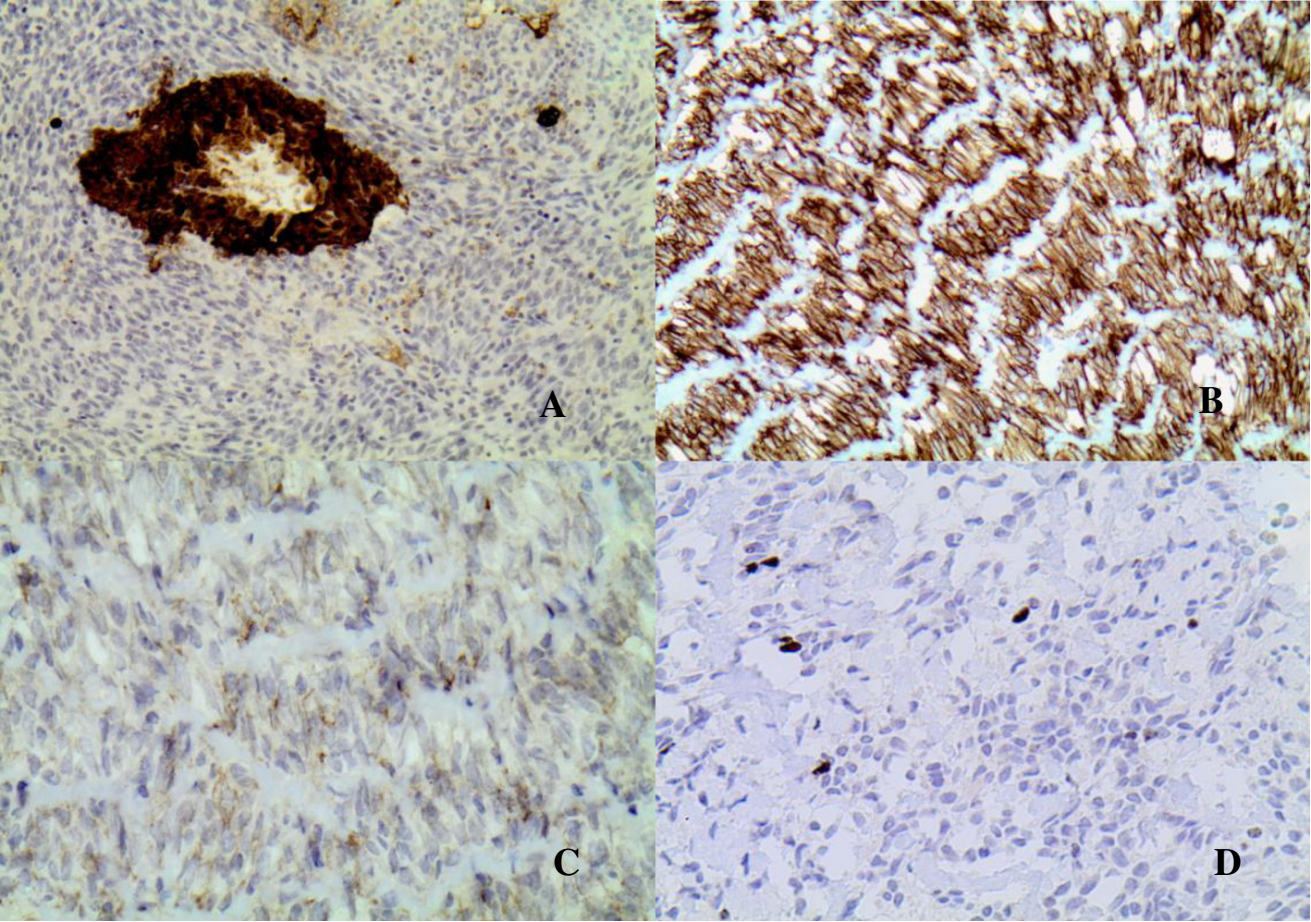
**Immunohistochemical studies.** (**A**) Epithelial component of the lesion is CAM5.2 positive. Sarcomatous stroma shows strong vimentin (**B**) and weak inhibin (**C**) immunoreactivity. (**D**) Sarcomatous component has a low Ki67 index (1% to 3%).

The epithelial component of the cysts was positive for CAM 5.2 antibody (Figure 3A). The stromal component in both pancreas and liver was strongly positive for vimentin (Figure 3B) and weakly positive for inhibin (Figure 3C). However, the spindle cells were negative for CD34, estrogen receptor, progesterone receptor, androgen, calretinin, S-100, CD117, melan A, chromogranin, and synaptophysin. The Ki67 index of stroma was low, 1% to 3% (Figure 3D).

## Discussion

Sarcomatous neoplastic lesions arising from the stroma of MCNs of the pancreas have been described in the literature (Table 1). The stromal component may be composed of bland, spindle cells with mild atypia and a small number of mitotic figures. These types of lesion have been described as ‘mucinous cyst adenoma with mesenchymal overgrowth or mural nodules’ [3,4]. In these tumors, the sarcoma-like foci are benign and do not affect the prognosis adversely. Other reported examples of MCNs include an associated malignant spindle cell stroma with characteristic features of malignant fibrous histiosarcoma [5,6], leiomyosarcoma [7], and undifferentiated pleomorphic high-grade sarcoma [8-10]. In some of these cases, the epithelial component was also malignant, so a diagnosis of carcinosarcoma was made.

**Table 1:** Summary of reported cases of mucinous cystic neoplasm of pancreas with sarcomatous component

**Reference**	**Demographic features**		**Epithelial component**	**Sarcomatous component**	
	**Age**	**Sex**	**Histology**	**Histology**	**Immunohistochemistry findings**
[8]	48	M	MCN, carcinosarcoma	Pleomorphism, high-grade atypia, atypical mitosis, necrosis	Vimentin (+)
					ER, PR (−)
				Liver and lymph node metastasis of carcinoma	S100 (−)
					CD34 (−)
					SMA (−)
[9]	70	F	MCN, invasive adenocarcinoma	Spindle cell sarcoma	Vimentin (+)
					Mucin 1 (+)
[10]	67	F	MCN, invasive adenocarcinoma	Pleomorphic sarcoma	Vimentin (+)
				Moderate to high-grade atypia	S100 (−)
				Atypical mitosis	c-Kit (−)
				Metastasis to liver	
[3]	30	F	MCN	MCN with mesenchymal overgrowth	ER, PR (+)
				Mild atypia, low number of mitoses	
				No metastasis	
[11]	48	F	MCN with atypia	MCN with sarcomatous stroma	Vimentin, MSA, SMA
					ER and PR (+)
	66	F	MCN with atypia	MCN with sarcomatous stroma	Vimentin,
				Moderate to high-grade atypia	MSA, SMA,
				Numerous mitoses	S100 (variable),
				Distant metastasis	ER and PR (+)
	67	M	MCN, invasive adenocarcinoma	MCN with sarcomatous stroma	Vimentin,
					MSA, SMA,
				Moderate to high-grade atypia	S100 (+)
					ER and PR (−)
				Numerous mitoses	
				Omental metastasis	
[5]	43	F	MCN	MFH	Vimentin, lysozyme, α1 antitrypsin
[4]	45	F	MCN	Pseudosarcomatous, mural nodules	CK, EMA, Vimentin

ER, estrogen receptor; MCN, mucinous cystic neoplasm; MFH, malignant fibrous histiocytoma; MSA, muscle-specific actin; PR, progesterone receptor; SMA, smooth muscle actin.

In three cases reported by Wenig *et al*. [11,12], stromal cells showed moderate to high-grade atypia, pleomorphism and increased numbers of mitotic figures. The sarcomatous component was angioinvasive. Two patients had metastatic lesions composed of sarcomatous lesion only. The epithelial component of one of these lesions showed features of invasive adenocarcinoma.

Our case differs from the previously reported literature by distant metastasis of only the stromal component, despite its morphologically benign features with bland spindle cells showing mild atypia, a low number of mitotic counts and a low Ki67 index. Both the sarcomatous stroma in the pancreas and the metastatic foci in the liver were strongly positive for vimentin and variably positive for inhibin. Estrogen receptor and progesterone receptor expression was not detected; this is not unlikely in MCN of pancreas arising in men.

## Conclusion

To the best of our knowledge, this is the first clinically MCN of the pancreas reported with distant metastasis of sarcomatous stromal component despite the relatively benign histologic features of the lesion. Although both epithelial and stromal components of the lesion are morphologically benign, the presence of a metastatic stromal focus in the liver renews the discussion that all pancreatic MCNs should be regarded as potentially malignant and that histology alone is not a definitive prospective indicator of behaviour [11,13]. According to most of the reported literature, the MCN with sarcomatous tumor behaves more aggressively; therefore, it is most likely that the sarcomatous component appears to be responsible for the malignant behaviour of these tumors and supersedes the epithelial component in terms of defining the aggressiveness of these tumors.

## Consent

The patient has given his consent for this case report to be published.

## Abbreviations

H & E: Hematoxylin and eosin; MCN: Mucinous cystic neoplasm; MFH: Malignant fibrous histiocytoma.

## Competing interests

The authors declare that they have no competing interests.

## Authors’ contributions

M W performed the surgery and completed the final paper. D G and V G are pathologists who evaluated the specimen. G A-gastroenterologist who diagnosed the tumor and helped in article writing. B A article writing and literature review. All author read and approved the final manuscript.
